# 5-HT and Intraplatelet 5-HT: a potential upstream regulator of YAP in liver regeneration

**DOI:** 10.1038/s12276-019-0324-1

**Published:** 2019-10-16

**Authors:** Chao He, Mimi Zhai, Bo Shu, Chaolin Deng, Li Li, Sushun Liu

**Affiliations:** 1 Department of General Surgery, the Second Xiangya Hospital, Central South University, Changsha, Hunan 410011 China; 2 School of Nursing, Changsha Medical University, Changsha, Hunan 410219 China; 3 Department of Gastroenterology and Hepatology, Mayo Clinic, Rochester, MN 55904 United States

**Keywords:** HIPPO signalling, Liver diseases

**To the Editor:**

We have read with interest the *Journal of Experimental & Molecular Medicine* article^1^. The author revealed that YAP and TAZ participated in liver regeneration. Additionally, liver regeneration was attenuated in the livers of YAP/TAZ-depleted mice. Thus, the author demonstrated that the Hippo pathway, especially the key components of the Hippo pathway, YAP and TAZ, was closely related to liver regeneration.

However, YAP and TAZ were　confirmed to be associated with liver regeneration. Whether YAP could be regulated and the identity of the upregulator of YAP are still confusing. Here, we noted that serotonin (5-HT) might be an upstream regulator of YAP. A study proved that serotonin receptors were related to liver regeneration^2^. Starlinger *et al*. also found that patients with lower 5-HT obtained poorer liver regeneration^3^. Additionally, our study proved that 5-HT affected the process of HCC by regulating YAP^4^. Another clinical study demonstrated that 5-HT and YAP were positively correlated in HCC patients^5^. Furthermore, we recently studied the relationship between 5-HT and YAP in liver regeneration. The results demonstrated that TPH1^−/−^ mice, lacking 5-HT, showed a worse regeneration ability and suffered more severe liver injury. Additionally, YAP expression was lower in TPH1^−/−^ mice then in WT mice, especially in the first three days after PH. Finally, we showed that the 5-HT-pERK-YAP axis might be involved in liver regeneration^6^. Thus, we hypothesized that 5-HT might be an upregulator of YAP in liver regeneration.

Platelets were the major carriers of serotonin. Thus, a factor that combines platelet and 5-HT levels might be more appropriate to evaluate the regulation of liver regeneration. Intraplatelet 5-HT (IP 5-HT) was a new factor that combined the effects of platelets and 5-HT. A study conducted by Starlinger et al. demonstrated that IP 5-HT was associated with liver dysfunction and morbidity. Additionally, the study proved that IP 5-HT was correlated with liver regeneration in humans. Patients with low IP 5-HT might suffer delayed liver regeneration^7^. A recent study conducted by Padickakudy et al. also indicated that preoperative IP 5-HT affected liver regeneration and tumor progression in a narrow window^8^. Thus, we speculated that IP 5-HT affected liver regeneration by regulating YAP and might be more appropriate than 5-HT to assess liver regeneration.

Although previous studies have indicated that serotonin and YAP both affect liver regeneration, the relationship between serotonin and YAP in liver regeneration has rarely been studied. No previous studies have explored the relationship between IP 5-HT and YAP in liver regeneration. Based on our study, 5-HT might be an upstream regulator of YAP in liver regeneration. Additionally, IP 5-HT might be more suitable to assess liver regeneration. We hypothesized that the IP 5-HT-YAP axis might be a stimulator during liver regeneration. Therefore, more basic and clinical studies are paramount to study the IP 5-HT-YAP axis and its potential mechanism in liver regeneration.
